# When Does the Human Embryonic Heart Start Beating? A Review of Contemporary and Historical Sources of Knowledge about the Onset of Blood Circulation in Man

**DOI:** 10.3390/jcdd9060187

**Published:** 2022-06-09

**Authors:** Jörg Männer

**Affiliations:** Group Cardio-Embryology, Institute of Anatomy and Embryology UMG, Georg-August-University Goettingen, D-37075 Goettingen, Germany; jmaenne@gwdg.de

**Keywords:** first heartbeat, human embryonic heart, human embryos, onset of blood circulation

## Abstract

The onset of embryonic heart beating may be regarded as the defining feature for the beginning of personal human life. Clarifying the timing of the first human heartbeat, therefore, has religious, philosophical, ethical, and medicolegal implications. This article reviews the historical and contemporary sources of knowledge on the beginning of human heart activity. Special attention is given to the problem of the determination of the true age of human embryos and to the problem of visualization of the human embryonic heart activity. It is shown that historical and current textbook statements about the onset of blood circulation in man do not derive from observations on living human embryos but derive from the extrapolation of observations on animal embryos to the human species. This fact does not preclude the existence of documented observations on human embryonic heart activity: Modern diagnostic (ultrasound) and therapeutic (IVF) procedures facilitate the visualization of early embryonic heart activity in precisely dated pregnancies. Such studies showed that the human heart started its pumping action during the fourth post-fertilization week. A small number of direct observations on the heart activity of aborted human embryos were reported since the 19th century, but did not receive much recognition by embryologists.

## 1. Introduction

From all the organs of our body, the heart and the brain have received an eminent position in the diverse philosophical, theological and biomedical concepts of human life that evolved in the Near East and Europe during the past 5000 years. Each of the two organs were proposed as the location of the soul or the source of the “vital principle” that defined personal human life [1]. Therefore, if we neglect the existence of concepts that attribute the soul or the vital principle to the whole body, we can roughly distinguish between heart-centered and brain-centered concepts of human life. Seeing either the heart or the brain as the source of the vital principle has great impact on the practical work of physicians. This is because the presence of heart or brain activity can be regarded as the indicator of life. The irreversible cessation of heart or brain activity then can be regarded as the defining sign of death of an individual human being. Up to the 1960s, the traditional legal definition of death, in Western countries, was the cessation of cardio-respiratory function. During the 1960s and 1970s, the medicolegal definition of death shifted from a heart-centered to a brain-centered definition of death, which had great impact on the development of transplantation medicine [2].

In analogy to the medicolegal definitions of death, the beginning of heart or brain activity may be regarded as defining features for the beginning of personal life [3]. These ideas also have consequences for the practical work of physicians since they have influence on the development of laws regulating abortion [4]. In the United States, for example, there is currently a tendency to define the beginning of personal human life by the onset of embryonic heart beating with the consequence of banning abortions after the time point of the first detection of the embryonic heart beating [5].

**Figure 1:**
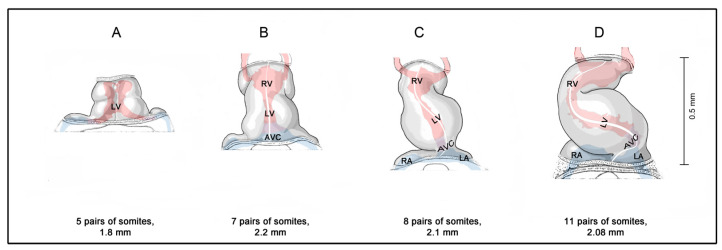
Morphogenesis of the human embryonic heart tube during Carnegie-stage 10. (**A**,**B**) depict early and late linear heart tube stages; (**C**) depicts a heart tube at the onset of cardiac looping; and (**D**) depicts a C-shaped heart tube. Hearts are shown in ventral views. The longitudinal white line indicates the original ventral midline of the heart tube. The shape of the endocardial lumen can be seen through the semitransparent wall of the hearts. It is indicated by red (ventricles, outflow tract, arteries) and blue (veins, atria, atrioventricular canal) colors. Drawings are based on Figures 18–24 shown in reference [10]. Abbreviations: AVC = atrioventricular canal; LA = embryonic left atrium; LV = embryonic left ventricle; RA = embryonic right atrium; RV = embryonic right ventricle.

In view of the eminent status of the heart in our thinking about personal human life, it is no wonder that, in my lectures of medical embryology, the students frequently ask me when the human embryonic heart starts beating. Up until last year, my answer to this question has corresponded to the current textbook knowledge, which says that the cardiovascular system is the first organ system to function and that the human embryonic heart starts beating at 21 to 23 days after fertilization [6,7,8]. During this short time period, human embryos are said to pass through stage 10 of the so-called Carnegie stages (CS) of human embryonic development, and their length is said to vary between 1.5 and 3 mm [9]. The heart of CS-10 embryos has a relatively simple form design, which resembles a tubular blood vessel. At the beginning of CS-10, the heart is a relatively short and straight tube aligned along the ventral midline of the foregut (Figure 1A). Up to the end of CS-10, the tubular heart undergoes a considerable lengthening and changes its 3-dimensional configuration from a straight to a C-shaped heart loop (Figure 1B–D).

It is obvious that a tubular embryonic heart mechanically cannot work in the same way as the mature four-chambered heart of human beings. Thus, if we use, in the context of the early embryonic heart activity, the term “heartbeat”, which is used to describe “*the regular movement that the heart makes as it sends blood around your body*” [11], we should be aware of the fact that we deal with a kind of heart movement that differs considerably from the movement of the mature four-chambered heart. Observations on animal embryos have shown that the pumping action of embryonic heart tubes is characterized by the cyclic generation of traveling mechanical waves sweeping from its venous to its arterial end. These waves were traditionally interpreted as peristaltic movements of the myocardial wall of the heart tube (for review see [12]). I should, furthermore, note that, in the embryological literature, the term heartbeat sometimes was used already for the first weak contractions of early embryonic cardiomyocytes [13,14,15,16]. These contractions appear as small, irregular twitches within circumscribed areas of the developing myocardium and do not generate coordinated movements of the developing heart that cause fluid flow. Calling these contractions heartbeats does not match with the above-mentioned everyday usage of the term heartbeat and, therefore, should be avoided.

**Figure 2:**
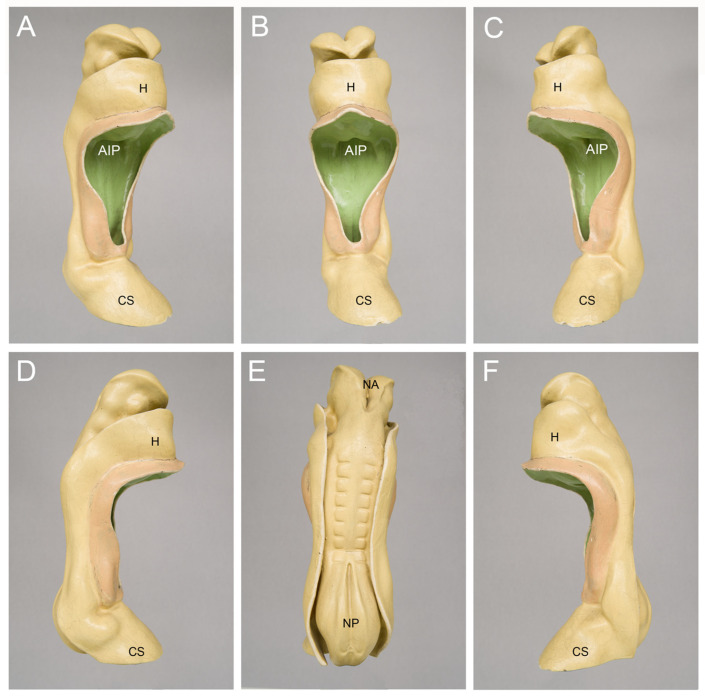
Outer morphology of a Carnegie-stage 10 human embryo as depicted by a historical three-dimensional model manufactured in the studio of Friedrich Ziegler in Freiburg/Breisgau, Germany. This embryo was described, for the first time, by the Swiss anatomist Auguste-Francois-Charles de’Eternod in 1896 and 1899 [17,18]. Pictures of this embryo or its heart were published in many textbooks, atlases and articles on human embryology during the 20th century. The embryo had a greatest length of 2.11 mm and seven pairs of complete somites + one pair of incomplete somites. The model is shown in right ventral (**A**), ventral (**B**), left ventral (**C**), right lateral (**D**), dorsal (**E**), and left lateral (**F**) views. Abbreviations: AIP = anterior intestinal portal; CS = connecting stalk; H = bulge of the body wall produced by the heart and pericardial cavity; NA = neuroporus anterior; NP = neuroporus posterior.

For the illustration of the general anatomy of CS-10 embryos, I usually show pictures of three-dimensional models of a well-known historical human embryo that has been described, for the first time, by the Swiss anatomist Auguste-Francois-Charles de’Eternod in 1896 [17] and 1899 [18] (Figure 2). The Eternod embryo had a greatest length of 2.11 mm and is best suited for illustrating an abridged and simplified version of the current textbook knowledge of the beginning of human heart activity, which says that the human embryonic heart starts beating around post-fertilization day 21, when the embryo passes through CS-10 and is about 2.1 mm long (21:10 = 2.1).

It should be noted here that the usage of the post-fertilization age is mainly confined to the community of embryologists and developmental biologists, which are especially interested in knowledge of the true age of human embryos. The date of fertilization of the human oocyte normally cannot be determined with high accuracy, since fertilization normally occurs within the body of the mother and is normally not associated with any physiological phenomenon that comes regularly to the attention of the mother. Physicians, therefore, determine the age of a pregnancy—the so-called “gestational age”—on the basis of a regular and visible phenomenon that normally does not escape the attention of a woman. This is the beginning of the last menstrual period [19]. Our current textbook knowledge of the relationship between menstruation, ovulation, and fertilization says that ovulation and fertilization normally occur approximately in the middle of an idealized menstrual cycle of 28 days. Thus, the gestational age is approximately 14 days older than the post-fertilization age. When expressed in terms of idealized gestational age, the human embryonic heart is said to start beating at 35 to 37 gestational days (sixth gestational week).

Having answered the above-mentioned students’ question in my lectures on medical embryology, I was recently confronted with a second students’ question, which asked for the sources of our current knowledge of the beginning of human heart activity. I think that this is a very nice students’ question since it forces us to scrutinize our current textbook knowledge and to think about the methods suitable for providing information on the beginning of heart activity in human embryos. The present review article summarizes the results of my research on contemporary and historical sources of knowledge of the beginning of human heart activity and, thereby, provides an answer to the challenging students’ question. This review is addressed not only to those working in the field of prenatal cardiogenesis but also to people outside of the area of biomedical sciences.

## 2. How Can We Clarify the Timing of the First Heartbeats in Human Embryos?

The ideal approach to clarify the timing of the first human heartbeats would be the direct observation of living human embryos during the critical phase of the fourth post-fertilization week (sixth gestational week). However, such an approach is not a trivial approach since fertilization as well as embryonic and fetal development of human beings normally takes place inside of the mother’s body, where the oocyte, the sperm, and the embryo are protected from direct visual observations. In other words, we normally do not know the exact fertilization date, nor can we see the embryo. If we want to obtain information on the beginning of human heart activity, by direct observations on living human embryos, we principally have to solve two practical tasks: (1) to make possible the exact determination of the post-fertilization age of the embryos to be examined; and (2) to make possible the visualization of their heart activity during the early stages of embryonic development.

## 3. Contemporary Sources of Knowledge

Today’s medical students may not see any problem in solving the two above-mentioned tasks. This is because modern medicine uses a wide spectrum of different diagnostic and therapeutic procedures, and two of these procedures seem to be ideally suited for solving the two tasks. These are: (1) assisted reproductive procedures, such as in vitro fertilization (IVF), which facilitate precise timing of fertilization in a very small population of human embryos since the late 1970s [20]; and (2) modern ultrasonographic imaging techniques, which make it possible to visualize, noninvasively and at relatively high spatial and temporal resolutions, human embryos and fetuses within the uterus from the beginning of the fourth week after fertilization (beginning of the sixth gestational week) up to the birth of the baby [21].

It appears that the currently most elegant design for a study intended to document the beginning of human embryonic heart activity seems to be an ultrasonographic examination of the embryonic heart activity in pregnancies resulting from assisted reproductive procedures. During the past 30 years, such studies were indeed conducted by several groups. The main purpose of these studies, however, was not to clarify the timing of the first heartbeat but was to provide reference curves for the changes in the embryonic heart rate in relation to the age and length of human embryos. Thus, in some of these studies, examinations have not started at the beginning of the fourth post-fertilization week, when the heart is said to start beating in some embryos but have started at the end of the fourth post-fertilization week (end of the sixth gestational week), when ultrasonographically detectable heart activity normally is present in every embryo [22,23]. It is obvious that such studies cannot provide information on the onset of embryonic heart activity. Only a relatively small number of studies, all published in the 1990s, started their examinations at the beginning of the fourth post-fertilization week and, therefore, could provide information on the onset of human embryonic heart activity [24,25,26,27,28,29,30,31,32,33]. Table 1 presents the age, greatest length, and heart rate of human embryos at the onset of ultrasonographically detectable heart activity as documented by these studies.

The analysis of the published data disclosed the following facts:

(1) Embryos of identical age can differ in the timing of the onset of ultrasonographically detectable heart activity. This means that human embryonic heart activity normally does not appear at a certain day of development but appears during a certain time slot. The length of this time slot varied between the studies listed in Table 1. The shortest time slot was 4 days [31,32] and the longest was 8 days [29]. Information on the length of the time slot was missed in three studies [27,30,33]. In these studies, the embryonic age was documented only for the earliest time point at which heart activity could be detected by transvaginal ultrasound. If the time slot for the onset of heart beating was defined by the data from normal continuing pregnancies, it was found that the appearance of the heartbeat after this time slot was associated with a higher chance of miscarriage [24,25]. The existence of time slots for the onset of embryonic heart activity corresponds to embryological data, which show that, among human embryos of the same coital age (=age determined after an isolated fruitful coitus), there is a great variability in the stage of development [34].

(2) In most studies, the reported time slot for the appearance of human embryonic heart activity was located at the end of the fourth and beginning of the fifth post-fertilization week [24,25,26,27,28,29] and, therefore, did not match with the time slot reported in contemporary textbooks of human embryology (21 to 23 days after fertilization). Only two studies documented the onset of heart beating at 20 to 23 days after fertilization and, thereby, provided data that matched with the textbook statements [30,31,32]. A third study documented the onset of heart beating on the 24th post-fertilization day, which was close to the textbook statements [33]. It should be noted here that the data from the last three studies, which were in accord with the textbook statements, were published in 1994, 1996, and 1998. The data from the other studies, which did not match with the textbook statements, all were published before 1994. In view of this fact, it is tempting to speculate that the mismatch between the data from the latter studies and the textbook statements may be explained by the usage of older ultrasound equipment, the resolutions of which did not facilitate the recognition of the embryonic heart activity before the end of the fourth post-fertilization week.

(3) In most of the studies listed in Table 1, the greatest length of human embryos, measured on the first day of ultrasonographically detectable heart activity, corresponded to the values obtained from post-mortem measurements on aborted CS-10 embryos (1.6 to 3 mm vs. 1.5 to 3 mm) [25,28,30,31,32] and, therefore, was in accord with the textbook statement that the human embryonic heart started beating at CS-10. Higher values (4 to 6 mm) were measured in three studies [26,27,29], while information on the embryonic length was missed in only a single study [33]. The higher values for the embryonic length were associated with heart rates that were markedly higher than those found in the studies in which the embryonic lengths were in the range of CS-10 embryos. This suggests that the deviations from the expected values of CS-10 embryos may be explained by the usage of ultrasound equipment, the resolutions of which permitted only a delayed detection of early embryonic heart movements. The textbook statement that the human embryonic heart starts beating during CS-10 is supported not only by the above-mentioned data from IVF pregnancies. It is also supported by data from ultrasound measurements on pregnancies resulting from physiological fertilization, which have shown that the smallest embryos in which heart activity could be detected had a greatest length of 2 mm [35,36]. It should be noted here, however, that the interpretations of the above-reported length values have some limitations: (1) To the best of the author’s knowledge, up to now, no study has addressed the question as to whether the values obtained from ultrasonographical in utero measurements of the greatest length of early human embryos correspond to the values that would be obtained by visual length measurements made on the same embryos after interruption of pregnancy. (2) The ultrasonographically determined length values may not be free of measurement errors.

With regard to ultrasonographic examinations of the activity of human embryonic hearts, I should finally note that the information on the age and length of human embryos, at the time of the first detectable heartbeats, was only a by-product of such studies. As stated above, the main purpose of such studies was to provide reference curves for the changes in the embryonic heart rate in relation to the age and greatest length of human embryos during the first trimester of pregnancy. This information is of great interest not only to physicians caring for pregnant women and their unborn children. It is also of interest to embryologists and developmental biologists working on the developing heart. This is because such data can be compared with historical and current data from non-human vertebrates that are frequently used as animal models for human embryonic development, such as the chick and mouse embryo. For unknown reasons, however, the data on the prenatal development of the heart rate did not find their way into contemporary textbooks of human embryology. I have, therefore, decided to include these data in the present review article.

**Figure 3:**
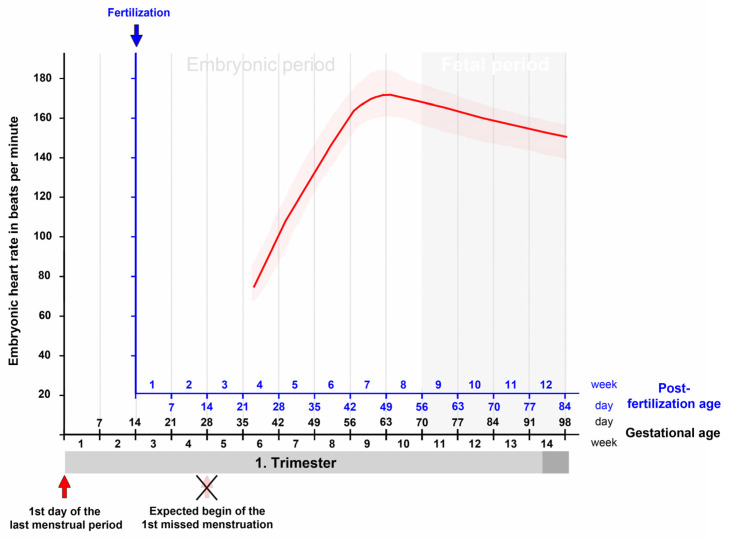
This graph depicts the typical changes in the embryonic/fetal heart rate in relation to the gestational as well as post-fertilization age as recorded by transvaginal ultrasonographic measurements during the first trimester. There is a rapid and almost linear increase in the embryonic heart rate from the onset of heart beating up to the end of the seventh post-fertilization week (ninth gestational week) followed by a moderate decrease up to the end of the first/beginning of the second trimester. Based on data from [30].

First trimester ultrasound examinations have consistently shown that the changes in embryonic heart rate follow a characteristic pattern [22,29,30,35,36,37]. This pattern is characterized by a first phase of a rapid and almost linear increase in the heart rate and a subsequent second phase of a moderate decrease in the heart rate (Figure 3). The first phase starts with the onset of heart beating and lasts up to the end of the seventh post-fertilization week. It coincides with the morphogenetic processes that transform the tubular embryonic heart into a four-chambered heart. Furthermore, during this phase, a significant correlation was found between the heart rate and the greatest length of the embryo [22,23,25,29,36,38]. The second phase starts at the beginning of the eighth post-fertilization week and lasts up to the end of the first/beginning of the second trimester. It coincides temporally with the structural maturation of the atrio-ventricular valves [39] and the maturation of the diastolic function of the ventricles [40].

**Figure 4:**
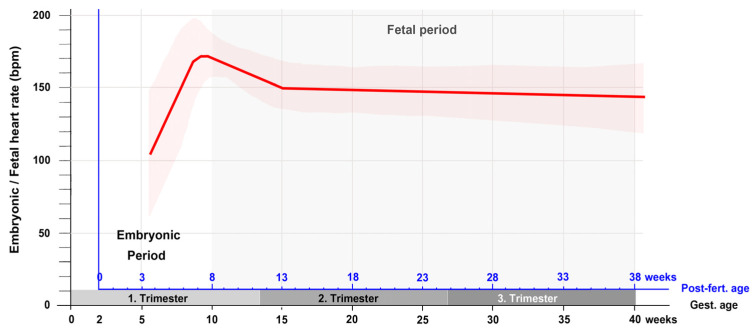
This graph depicts the typical changes in the embryonic/fetal heart rate in relation to the gestational as well as post-fertilization age as recorded by ultrasonographic measurements during all periods of pregnancy. Note that after the first trimester, there is only a slow linear decrease in the heart rate up to the birth of the baby. Based on data from [41,42]. Abbreviations: bpm = beats per minute; Gest. = Gestational; Post-fert. = Post-fertilization.

If the heart rate data from the first trimester are complemented by measurements from the second and third trimester, it is found that the phase of moderate decrease in the heart rate is followed by a phase of a slow decrease that lasts up to the birth of the baby [41,42]. A curve depicting the changes in the embryonic and fetal heart rate during all periods of prenatal development is shown in Figure 4.

If this figure is compared with corresponding figures from chick (Figure 1 in [43]) and mouse (Figure 1 in [16]) embryos, it becomes apparent that the patterns of prenatal changes in the heart rate are very similar between humans and chicks. Mice, on the other hand, do not show a decrease in the heart rate during the fetal period of development. In these animals the prenatal heart rate does not only increase during the embryonic period (mouse post-fertilization days 1 to 14.5) but also increase during the fetal period (mouse post-fertilization days 14.5 to 21) as well as during postnatal heart development until the animals reach adulthood.

## 4. Historical Sources of Knowledge

### 4.1. Knowledge about the Developmental Stage of Human Embryos at the Onset of Heart Beating Can Be Traced Back in Time up to the Late 19th Century

The above-mentioned ultrasound studies from the past 30 years have provided some data that seem to be in accord with the current textbook statements on the onset of heart beating in human embryos. However, for two reasons, these sonographic data cannot form the basis of our current textbook knowledge:

(1) The current textbook statements on the timing of the first heartbeats can be traced back in time up to the appearance of the first edition of Patten’s “*Human Embryology*” in 1947 [44]. At that time, IVF of the human ovum was in its earliest steps of experimental testing [20] and ultrasound made its very first steps into usage as a diagnostic tool in medicine but had not started its career in obstetrics and gynecology, which began in 1958 [45].

(2) Current ultrasonographic imaging techniques facilitate the determination of the size and the detection of the heart activity of early human embryos. However, they do not facilitate the detailed analyzes of the morphology of early human embryos, which is needed for the correct staging of these embryos according to the CS of human embryonic development [46,47]. Thus, the information that human heart activity starts during CS-10 cannot come from ultrasonographic examinations of living human embryos within the uterus.

The CS of human embryonic development were defined on the basis of data from post-mortem examinations of a large number of embryos from the collection of human embryos originally housed in the Department of Embryology at the Carnegie Institution of Sciences, Washington. These embryos derived from spontaneous abortions, forensic sections, or surgical interventions (e.g., treatment of ectopic pregnancies, removal of an uterus myomatosus). The developmental period defined as CS-10 was first described in 1957 (at that time called “*age group X*” or “*horizon X*”), and it was already in this paper that the onset of cardiac pumping activity was assigned to this stage of development and that the age of CS-10 embryos was estimated to be 22 ± 1 days after fertilization [14].

Before the publication of Patten’s embryology, information on the early function of the embryonic cardiovascular system was rarely provided in textbooks or handbooks of human embryology. Embryologists of that time period were primarily interested in uncovering the dynamically changing anatomy of human embryos. However, if such information was provided, it consisted of descriptions, illustrations, and statements that already attributed the beginning of heart beating to human embryos that nowadays would be classified as belonging to CS-10 or CS-11 [13,48,49]. The accompanying statements on the age of these embryos, however, differed markedly from those found in the embryological literature since the 1940s. Two examples should illustrate this finding.

(1) In his famous “*Lehrbuch der Entwicklungsgeschichte des Menschen*” (textbook on the ontogenesis of human beings) from 1898, the German anatomist Julius Kollmann stated that “*Schon bei menschlichen Embryonen von ungefähr 2 mm Länge besteht ein Kreislauf, der durch die Kontraktionen des Herzschlauches unterhalten wird*.” (“A circulation, driven by contractions of the heart tube, is already present in human embryos of approximately 2 mm length.”) [48] (pp. 445–446). His descriptions and illustrations of such embryos matched with the morphology of CS-10 and CS-11 embryos but the age of these embryos was reported to be 12–14 days after fertilization [48] (pp. 196–206).

(2) In the first volume of his monograph on the “*Anatomie menschlicher Embryonen*” (anatomy of human embryos) from 1880 [49], Wilhelm His senior described the anatomy of human embryos from the first month of development. He distinguished 10 developmental stages within this period. With regard to the heart of embryos from his stages 6 and 7, which nowadays would be classified as CS-11 embryos, he stated: “… *das Herz ist als Schlauch angelegt und, laut den Erfahrungen an Thierembryonen bereits thätig*” (“ … the heart is formed as a tube and, according to observations made on animal embryos, is actually working.”) [49] (p. 155). The age of these embryo was reported to be 16–18 days after fertilization [49] (p. 168).

### 4.2. Historical Changes in the Statements on the Age of Human Embryos

The reason for the above-described deviations from the current textbook statements on the post-fertilization age of CS-10 and CS-11 embryos lies in the historical evolution of our concepts of the relation between menstruation, ovulation, and fertilization, which determined most methods used for the estimation of the post-fertilization age of human embryos. Without knowledge of this history, we cannot correctly evaluate statements on the true ages of human embryos made in historical as well as current publications.

My historical overview starts with a method for age estimation that was introduced into the scientific discipline of human embryology by the above-mentioned anatomist Wilhelm His senior (*1831–1904†), who may be regarded as the founder of modern human embryology [50]. Wilhelm His favored calculations of the true age of human embryos that were based on a concept of the relation between menstruation, ovulation, and fertilization that was introduced by his colleague Karl Bogislaus Reichert (*1811–1883†) in 1873 [51]. According to this concept, ovulation and fertilization should occur 2 to 3 days before the expected beginning of the first missed menstrual bleeding. This meant that, with regard to an idealized menstrual cycle of 28 days, the “Reichert-His ages” could be calculated by subtracting 25 to 26 days from the known gestational ages. With regard to human embryos with the morphological features of CS-10, the proposed Reichert-His age was 12 to 14 days [48,52,53]. The estimation of the post-fertilization age of human embryos according to the so-called “Reichert-His convention” replaced the traditional reckoning from the end of the last menstrual bleeding and dominated the young scientific discipline of human embryology between 1880 (appearance of the first volume of His’s “*Anatomie menschlicher Embryonen*” [49]) and 1910 (appearance of the German edition of Keibel’s and Mall’s “*Manual of Human Embryology*” [54]).

During the first two decades of the 20th century, observations were made that were at variance to the Reichert-His concept of the relation between ovulation, fertilization and menstruation [55,56,57,58,59]. As a consequence, the Reichert-His convention came out of use. The new data suggested that ovulation and fertilization of the human oocyte took place in the intermenstrual interval. There were, however, various opinions about the time relation between menstruation and ovulation. Franklin Paine Mall (*1862–1917†), the founder and first director of the Department of Embryology at the Carnegie Institution for Sciences, for example, has suggested that ovulation and fertilization may take place about 11 days after the onset of the last menstrual bleeding [58,59]. Other researchers thought that ovulation and fertilization might occur at any time of the intermenstrual interval [55].

Up to the 1930s, evidence had accumulated that ovulation and fertilization of the human oocyte takes place at a time near the middle of the menstrual cycle, thus preceding the next menstrual bleeding for about 14 days [60,61,62]. This meant that, with regard to an idealized menstrual cycle of 28 days, the post-fertilization age could be calculated by subtracting 14 days from the known gestational age. Since the 1940s, calculations according to the concept of “mid-cycle ovulation” were used for the age estimation of human embryos by several researchers. In view of the well-known variability in the length of the menstrual cycle, however, it is no wonder that the results of such calculations varied between different studies. In Hamilton’s, Boyd’s and Mossman’s textbook on “*Human Embryology*” [63], for example, the calculated post-fertilization age of CS-10 embryos was 20 to 23 days, which fits to the current textbook statements. Based on data from the Kyoto-Collection of human embryos, on the other hand, the post-fertilization age was calculated as 26 to 28 days, which is 5 days older than the age proposal given in current textbooks [64].

Calculating the real age of human embryos according to methods based on the menstrual history could not provide exact information. Therefore, George Linus Streeter (*1873–1948†), the second director of the Department of Embryology at the Carnegie Institution for Sciences, chose a different approach for the determination of the real age of human embryos. Studies on rhesus monkeys (*Macaca mulatta*) had shown that it was possible, in this primate species, to obtain embryos of known ovulation age at any desired stage of morphological development. It was, furthermore, found that with regard to size and external morphology, macaque and human embryos were nearly identical during the first 6 weeks of development. Streeter and co-workers, therefore, have suggested that, “*… in respect to both size and form, the most accurate way to estimate the age of a human embryo during the early weeks is to compare it with the macaque embryos of known age …*” [65] (p. 45). The “Streeter ages” of human embryos became integral components of the CS of human embryonic development [9,14,66], and, thereby, found the way into textbooks of human embryology. The Streeter age of CS-10 embryos was determined as 21 to 23 days [14].

The historically youngest approach for the estimation of the age of human embryos was introduced by Ronan O’Rahilly and his wife Fabiola Müller in 2010 [47]. Ronan O’Rahilly (*1921–2018†) was the director of the Carnegie Collection of Human Embryos from 1974 to 1990. On the basis of Streeter’s horizons [14,66], he and his wife had developed the CS of human embryonic development. With regard to the age determination of human embryos, O’Rahilly and Müller have used a clinically proven method, which correlates size measurements on specimens from the Carnegie Collection with reference growth curves derived from ultrasonographic examinations of exactly dated pregnancies (conceived after IVF). The post-fertilization age of CS-10 embryos estimated by this approach was 28 to 30 days, which is 7 days older that the age proposal given in current textbooks. Table 2 summarizes the historically most important methods used for the estimation of the true ages of human embryos and the resulting age proposals for CS-10.

### 4.3. Extrapolating Observations on Animal Embryos to the Human Species

Having clarified the reasons for the above-mentioned historical differences in the textbook statements on the post-fertilization age of human CS-10 embryos, the question remains as from which sources embryologists had their knowledge about the morphological stage at which the human heart normally starts beating. Were it direct observations on living human embryos outside of the uterus, or were it observations on animal embryos? The answer seems to be clear since it is commonly stated by embryologists, since the 1930s, that no direct observations on the onset of circulation have been made in human embryos [44,69,70,71]. Therefore, information on the timing of the first human heartbeat usually derives from the extrapolation of observations on animal embryos to the human species [16,44,49,69,71,72]. The most suitable animal models for the ontogenesis of the human embryonic heart function may be embryos from primate species such as the above-mentioned rhesus monkey *Macaca mulatta* [65]. Unfortunately, however, the original Carnegie report on the developmental stages of these macaque embryos did not provide any information about the morphological as well as functional development of the heart [65]. Moreover, since the publication of this report, only a single paper was published, which provided ultrasonographical data on the in utero development of the embryonic and fetal heart activity of rhesus monkeys [73]. It was reported that ultrasonographically detectable heart activity of rhesus monkey embryos started at 21 days after the middle of a successful 5-day mating period of the animals. More precise information on the age and developmental stage of macaque embryos at the onset of blood flow could not be found in the literature on the embryonic development of non-human primates. In view of the lack of exact data from primate embryos, information on the timing of the first human heartbeat usually derives from the extrapolation of observations on non-primate animal embryos to the human species. This method is not a trivial approach since the relation between morphological and functional development of the heart differs markedly across vertebrate species [74]. This phenomenon is named heterochrony. It is a well-known fact that the relation between the onset of blood flow and the morphological stage of the embryonic heart tube shows species-specific differences. The initially straight heart tube of vertebrate embryos is a single median structure that forms by the union of materials from bilaterally paired heart fields in front of the developing foregut [75]. In some mammalian species, such as rabbits [76] and rats [69], myocardial differentiation and the onset of pumping activity precede the union of the paired heart fields. In these species, a bilateral pair of endocardial tubes, surrounded by a contracting myocardial mantle, is formed within the two heart fields shortly before the latter start their union along the ventral midline of the foregut to form the single median heart tube. In this situation, the median heart tube seems to arise from the union of a pair of actively pulsating blood vessels, which have been named “lateral hearts” [69]. In other vertebrate species, such as chicks [77] and mice [78], however, the onset of circulation occurs at median heart tube stages, which means after the heart fields have started their union [79,80,81,82]. In view of these findings, the question arises as to whether the onset of the human embryonic heart activity follows the “rabbit” or the “chicken/mouse” mode of early cardiogenesis. In the former case, the first heartbeat would be expected to occur at the beginning CS-9 (before union of the paired heart fields). The proposed post-fertilization age of CS-9 embryos is 20 ± 1 days [9]. In the latter case the first heartbeat would be expected to occur during CS-10 or later (after the heart fields have started their union). Studies on human embryos have shown that the development and mode of union of the human embryonic heart fields resembles that observed in chick and mouse embryos [10,70]. Therefore, these animal embryos may be regarded as the most suitable non-primate models for the ontogenesis of the human embryonic heart activity. In the following, a short summary is given on our current knowledge about the beginning of heart activity as derived from observations on chick and mouse embryos [15,16,79,80,81,82,83].

The embryonic heart is not a machine that begins its pumping action by pushing a start button. Observations on animal embryos have shown that the onset of embryonic blood circulation is a gradual process [79,80,81,82]. It starts with spontaneous electric activity (irregular action potentials) [83] and asynchronous Ca^2+^ oscillations [15,16,80] within discrete foci or single cells of the developing myocardium. The onset of electric activity and Ca^2+^ oscillations is soon followed by the formation of myofibrils and the onset of myocardial contractions [15,16,80,83]. The first contractions of the immature myocardial cells appear as small, irregular twitches within circumscribed areas of the developing myocardium [15,16,79,80,83]. These contractions do not generate a coordinated movement of the myocardial mantle of the developing endocardial tube, which is needed for the generation of a directed fluid flow within the vascular network of the embryonic cardiovascular system. The initially weak contractions become stronger, and the initially irregular pattern of myocardial contractions soon develops into a coordinated, peristaltic-like motion of the heart tube that, firstly, generates directed flow of intravascular fluid devoid of erythrocytes (plasma) and later drives the continuous circulation of erythrocytes through the vascular network of the embryo and the gestational sac [79,80,81,82].

Extrapolating the observations on mouse embryos to the human species suggest that spontaneous action potentials and weak myocardial contractions commence at the end of CS-9, when the heart fields have started their union to form a short linear heart tube. Unidirectional plasma flow then becomes established during CS-10, when the linear heart tube elongates and starts its transformation into a C-shaped heart loop. The continuous circulation of erythrocytes, dispersed in the plasma, may start at the transition from CS-10 to CS-11, when the heart has reached the phenotype of a C-shaped heart loop and is connected to the dorsal aortae via the first pair of pharyngeal arch arteries.

## 5. A Search for Published but Unknown Ex Utero Observations on Human Embryonic Heart Activity

The rational for the above-described usage of animal observations in clarifying the timing of the first heartbeat in man is based on the statement that no direct observations on the onset of circulation have been made in human embryos [44,69,70,71,72]. It is a question, however, as to whether this statement is really true. Did more than 200 years of research on aborted human embryos really never provide any documented observation on a beating human heart at the onset of circulation? To answer this question, I have systematically checked the embryological as well as non-embryological literature from the past 200 years.

### 5.1. The Embryological Literature (Journals and Books on Embryology, Anatomy, Teratology)

#### 5.1.1. Reports on CS-10 Embryos

My search for documented observations on human embryonic heart activity started with a check of the published case reports on CS-10 embryos. Among these reports, there was indeed not a single one that documented direct observations of heart beating. This finding can be explained, in part, by the fact that most embryo specimens were obtained from physicians residing at places that were remote from the laboratories of the embryologists. For reasons of good tissue preservation, these specimens were already placed in fixing fluids (e.g., formalin, Bouin’s solution) before they were sent to the embryologists. Only a few specimens came from physicians who worked close to the institutions of the embryologists and, therefore, could be sent to the laboratory in a fresh state [84,85,86,87,88]. This principally should have permitted direct observations on living embryos. In most of the fresh specimens, however, the embryo could not be observed in a vital state since it was embedded in an intact chorionic sac that was placed in fixing fluid immediately after arrival in the laboratory [84,86,87]. There were only two fresh specimens where the chorionic sac was opened by the embryologists prior to fixation and, thereby, facilitated a direct view on the embryo. But even for these specimens no records of embryonic heart activity were documented in the papers reporting on them [85,88]. Table 3 summarizes the most important information given in the published case reports on human CS-10 embryos.

#### 5.1.2. Reports and Studies on Collections of Human Embryos

I next checked all available reports or studies on general aspects of human embryo collections (e.g., provenance, conditions and handling of specimens). Among these articles, there were two reports that provided information on heart activity [108,109]. Both articles reported on the Carnegie Collection of Human Embryos. The first was published in 1911, when the collection comprised 533 specimens [108], and the second was published in 1921, when the collection comprised 2500 specimens [109]. In these reports Franklin P. Mall stated that in several instances human embryos or fetuses were brought to his laboratory still alive, and that this fact has made it possible to obtain complete vascular injections with India ink while the hearts of the specimens were still beating. The 1921 report provided only a single example for such a specimen. This embryo had a greatest length of 21 mm, which is above the range of length values reported for CS-10 embryos. In the 1911 report, a catalogue of all specimens was provided that were collected up to that time. Among the 533 specimens listed in this catalogue, there were 22 human embryos and fetuses marked as “injected” specimens. In most of these specimens the injection was done by Max Brödel (*1870–1941†], a skillful medical artist [110], who had his laboratory in the Gynecological Department of the Johns Hopkins Hospital in Baltimore, Maryland. The smallest injected specimen was 11 mm long (No. 353); it was classified as a CS-17 embryo some decades later [111]. The largest specimen was a fetus, which had a greatest length of 96 mm (No. 484). Injected embryos from the Carnegie Collection were used in studies on the development of the vascular supply of various organs, such as the brain and the heart [111,112,113]. Pictures of some injected embryos can be found in the second volume of Keibel’s and Mall’s *Manual of Human Embryology* [114]. The above-mentioned 11 mm embryo, for example, is shown in Figure 469, on page 687 of the manual. Thus, with regard to reports on human embryo collections, I can summarize that a few of these reports have provided evidence for the presence of heart activity at advanced stages of embryonic development (CS-17 and older). However, these reports did not provide any documented observation on heart activity at developmental stages usually associated with the onset of blood circulation (CS-10, or CS-11). Table 4 summarizes the most important data provided in the reports or studies on human embryo collections that had been checked for information on embryonic heart activity.

#### 5.1.3. Embryological Studies Focusing on Human Embryonic Hearts

My analysis of the embryological literature ended with a check of published studies on human embryonic hearts. Thereby, only those studies were considered in which hearts from CS-10 embryos were included. Among the nine studies fulfilling this criterion, only a single study provided indirect evidence for the presence of heart activity. This study used embryos from the Carnegie Collection and it was reported that ten of the specimens had been injected [111]. The developmental stages of these specimens ranged from CS-17 to 22. Thus, corresponding to the above-described observations, one can say that embryological studies on human embryonic hearts have not provided any documented observation on heart activity at developmental stages usually associated with the onset of blood circulation. Table 5 summarizes the most important data provided in the nine studies on human embryonic hearts that had been checked for information on heart activity.

### 5.2. The Non-Embryological Literature

I finally checked the “non-embryological” literature for the presence of documented *ex utero* observations on the heart activity of human embryos or fetuses. When I speak of “non-embryological” literature I mean journals or books of physiology, gynecology and obstetrics, reproduction medicine, cardiology, and other disciplines not primarily related to the morphogenesis of human embryos after the third post-fertilization week (after the fifth gestational week). In contrast to the situation in the above-described embryological literature, I found that *ex utero* observations on the heart activity of human embryos or fetuses were relative frequently reported here since the 19th century. Among these observations one can roughly distinguish four different kinds of reports: (1) Case reports on incidentally obtained specimens. Such reports mostly focused on the fact that the embryos or fetuses were able to survive the spontaneous interruption of the pregnancy for a relatively long time span [127,128,129,130,131,132,133]; (2) Reports on electrocardiographic studies on embryos or fetuses [134,135,136,137]; (3) Reports on electrophysiological studies on isolated heart specimens or cardiomyocytes [138,139,140,141]; (4) Reports on experimental studies, which tested the effects of catecholamines, acetylcholine, and other substances on the activity of isolated heart specimens [142,143,144,145,146]. Most of the latter studies were conducted in order to obtain information on the functional maturation of the nervous control of the heart activity. Table 6 provides a list of reports on human embryonic or fetal heart activity published in the non-embryological literature.

From the 20 reports listed in this table, there were nine, which reported on embryonic specimens [127,131,132,133,135,136,138,139,145,147]. From these nine reports, three should be highlighted here since they report on interesting observations made on embryos at an early stage of heart development.

The first report was made in 1877 [127] by the well-known German physiologist Eduard F.W. Pflüger (*1829–1910†). Pflüger reported on a young human embryo, which he had obtained, enclosed in a slightly damaged chorionic sac, from a midwife shortly after spontaneous abortion. He received the fresh specimen at nightfall which, unfortunately, prevented its immediate examination under a standard light microscope, which, at that time, used sunlight or ordinary daylight for the illumination of the objects. Pflüger, therefore, stored the unopened chorionic sac between two watch glasses overnight in a cold room. He opened the chorionic sac in a warm room, at the next morning, and thereby found a slightly damaged embryo with a widely open communication between the developing gut and the yolk sac, a neural tube at an advanced stage of closure, and an s-shaped heart tube close to the head. To his great surprise, he noted that, coincident with the warming up of the specimen, the heart tube started to contract at intervals of 20 to 30 s. He described the character of these contractions, which continued for more than 1 h, as resembling those he had observed in chick embryos at this stage of development. Pflüger did not provide any information on the menstrual age or size of the embryo but estimated the age of his embryo, on the basis of the size of the chorionic sac, as 18 to 20 days. The morphological features reported by Pflüger suggest that his embryo was at CS-11. The Pflüger report on human embryonic heart activity was the first of its kind in history and became a relatively well-known observation among the community of developmental physiologists [72,148,149] but did not receive much attention by embryologists.

The second report was made by William F. Armann a physician from Ritzville, Washington, USA. It was published twice. A German version was published in the *Archiv für Gynäkologie und Geburtshilfe* in 1908 [131], and an English version appeared in the *American Journal of Obstetrics and Diseases of Woman and Children* in 1913 [132]. The embryo described in these reports had survived, for a short time, spontaneous abortion at 6 weeks after the last menstrual period. It was found in a physically intact gestational sac, which was as large as a hazelnut. Its body had a greatest length of 2.5 mm and its tubular heart showed regular beating at a frequency of 90 beats per minute. Regular heart pulsations continued for at least 15 min after opening of the chorionic sac. Armann reported that he had compared his embryo with the figures shown in Kollmann’s embryology [48] and had found that it corresponded to a 2.5 mm embryo of about 14 days (estimated age according to Reichert-His!). Unfortunately, he did not report which of Kollmann’s figures he had judged as a corresponding figure to his embryo. It is most likely that it was Figure 119, which showed the 2.5 mm “Bulle” embryo. The “Bulle” embryo belonged to Kollmann’s collection of human embryos and was first described in 1889 [150]. It is found in the lists of historical CS-11 embryos published by Streeter [66], and O’Rahilly and Müller [9]. The Armann embryo, therefore, may be classified as a CS-11 embryo. A somewhat puzzling finding is the fact that the two versions of Armann’s report present absolutely identical stories except for a single detail, which is the date of the observations (May 1906 vs. May 1912). This fact may cast some doubt on the correctness of Armann’s story. I therefore, have compared Armann’s information on the size of the chorionic sac (“large as a hazelnut” = 17–19 mm), the size of the yolk sac (“large as a lentil” = 3–8 mm), the greatest length of the embryo (2.5 mm), and the heart rate (90 bpm) with recent ultrasonographic measurements from IVF pregnancies [23]. This analysis disclosed that all of Armann’s data were in the range of the values measured in utero at post-fertilization day 28 (gestational day 42). I, therefore, think that the Armann reports on early embryonic heart activity are not fake reports. It is a question why these reports were noted only by a few gynecologists [133,151,152,153,154] but had escaped the attention of developmental physiologists and embryologists for more than 100 years.

**Figure 5:**
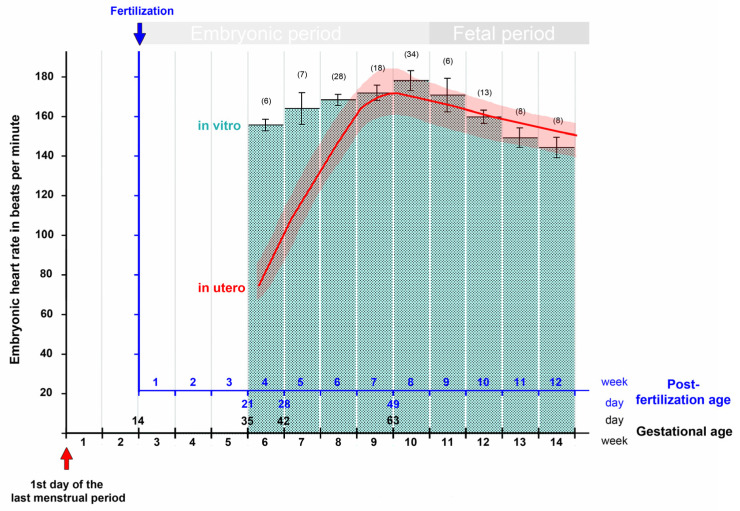
This graph depicts the changes in the contraction rate of human embryonic and fetal hearts during the first trimester of pregnancy as recorded (1) in vitro on isolated heart specimens obtained from aborted embryos and fetuses (data from [147]; mean values ± S.E.; number of cases in parentheses); and (2) in utero by transvaginal ultrasonography (data from [30]). Note that, during the first 3 weeks of heart activity, the in vitro values are markedly higher than those measured in utero. From the seventh post-fertilization week (ninth gestational week) onward, however, in vitro and in utero values do not differ markedly from each other.

The third report came from a group of Hungarian gynecologists. The members of this group were interested in uncovering the ontogenesis of the parasympathetic nervous control of the human heart activity. For this purpose, they conducted systematic in vitro studies on the hearts of human embryos and fetuses obtained from legal terminations of pregnancies. In one of these studies, the changes in the intrinsic contraction rates of isolated heart specimens were documented for the time period between the end of the fifth and end of the 15th week of gestation [147]. The isolated heart specimens were cultured in modified Locke’s solution at 37 °C, and the heart rates were recorded via surface electrocardiograms. Measured values were documented for ten successive age groups of 1 week (Figure 5). In this study, regular heartbeats were already recorded in the specimens from the youngest age group, which comprised hearts from embryos at a gestational age of 5 weeks. This observation principally fits to our current textbook statements on the onset of human embryonic heart activity. The relatively rough estimation of the age of the specimens, however, does not allow precise timing of the first heartbeats. Moreover, the authors did not provide any information on the morphology of the embryos, which could be used for staging according to the CS of human embryonic development. Thus, the exact age as well as the CS of the specimen described in this study must remain an open question. Apart from this shortcoming, the report from the Hungarian group provides an interesting finding. If the in vitro data are compared with the in utero data from precisely dated pregnancies, it is noted that, during the first 3 weeks of heart activity, the in vitro values are markedly higher than those measured in utero. From the seventh post-fertilization week (ninth gestational week) onward, however, the in vitro and in utero values do not differ markedly from each other (Figure 5). This observation suggests that, for human heart specimens older than the seventh post-fertilization week, the environmental conditions of the in vitro culture may have sufficiently mimicked the physiological environmental conditions, while for younger specimens the environmental conditions of the in vitro culture may have deviated significantly from the normal ones. Thus, the characteristic peak of the embryonic heart rate at the transition from the seventh to the eighth post-fertilization week (ninth to tenth gestational week) may be a landmark for a marked change in the environmental conditions of our prenatal life. In this regard, it may be of interest that the initially low oxygen concentration in the intervillous spaces of the human placenta shows a rise between the ninth and fourteenth gestational weeks [155], suggesting that the oxygen concentration may be one of the suspected environmental factors. Studies on isolated human embryonic hearts indeed have shown that a reduction in oxygen in the culture medium causes a slowing of the ventricular contraction rate [139]. Future studies are needed to test this hypothesis.

## 6. Summary and Conclusions

The onset of embryonic heart beating may be regarded as the defining feature for the beginning of personal human life. This concept has influence on the development of laws regulating abortion. Answering the question as to when the human embryonic heart starts beating, therefore, has consequences for the practical work of physicians.

The present review article reports on historical and contemporary sources of knowledge on the beginning of heart activity in human embryos.

Prenatal development starts with the fertilization of the oocyte. In human beings, fertilization as well as embryonic and fetal development normally takes place inside of the mother’s body. Thus, information on the age and developmental stage of human embryos at the onset of heart activity can be obtained only by solving two practical tasks: (1) to make possible the visualization of the morphology and heart activity of human embryos during the early stages of prenatal development; and (2) to make possible the exact determination of the post-fertilization age of the embryos to be examined.

Knowledge of the morphological development of human embryos was traditionally derived from post-mortem studies on human embryos obtained from spontaneous abortions, forensic sections or therapeutic interventions. The age of these embryos was traditionally estimated on the basis of the menstrual history or by matching the specimens against macaque embryos of known ovulation age at the same stage of morphological development.

It is generally reported in the embryological literature that, in the past, no direct observations have been made on the beginning of blood circulation in living human embryos. Therefore, information on the onset of the pumping activity of the human embryonic heart was traditionally derived from the extrapolation of observations on living animal embryos to the human species. This information formed the basis of our current, as well as historical, textbook knowledge about the onset of circulation in man.

Observations on animal embryos have shown that the onset of embryonic blood circulation is a gradual process, which can be subdivided into four subsequent steps: (1) Appearance of spontaneous electric activity and Ca^2+^ oscillations within the developing myocardium. (2) Onset of weak myocardial contractions that appear as small, irregular twitches within circumscribed areas of the developing myocardium. These early contractions do not generate a coordinated movement of the myocardial mantle of the developing endocardial tube, which is needed for the generation of a directed fluid flow within the embryonic cardiovascular system. (3) Emergence of a coordinated, peristaltic-like motion of the heart tube that, firstly, generates directed flow of intravascular fluid devoid of erythrocytes (plasma) and later (4) drives the continuous circulation of erythrocytes through the vascular network of the embryo and the gestational sac.

Extrapolating the observations on animal embryos to the human species suggest that spontaneous action potentials and weak myocardial contractions commence at the end of CS-9, when the heart fields have started their union to form a linear heart tube. Unidirectional plasma flow then becomes established during CS-10, when the linear heart tube elongates and starts its transformation into a C-shaped heart loop. The continuous circulation of erythrocytes, dispersed in the plasma, may start at the transition from CS-10 to CS-11, when the heart has reached the phenotype of a C-shaped heart loop.

The term “heartbeat” is used to describe “*the regular movement that the heart makes as it sends blood around your body*” [11]. The above-mentioned observations suggest that, with regard to the embryonic heart, we should not speak of a beating heart before coordinated regular movements of its walls generate a unidirectional fluid flow within the vascular network of the embryonic cardiovascular system. In human embryos this functional state seems to be reached during CS-10.

Based on data from macaque embryos, the post-fertilization age of human CS-10 embryos was estimated as 21 to 23 days (idealized gestational days 35 to 37) in 1957. This information is usually provided in textbooks of human embryology. During the past 60 years, this age proposal has been questioned several times by data from studies on collections of aborted human embryos. In these studies, embryonic ages were calculated by subtracting 14 days from the reported gestational ages and it was noted that the estimated ages were 4 to 5-days-older than those obtained from the comparison of human embryos with macaque embryos. Based on new measurements of the length of historical specimens from the Carnegie Collection, the ages assigned to each CS were recently determined from reference growth curves derived from ultrasonographic in utero studies on precisely dated pregnancies (conception by IVF). It is proposed that the post-fertilization age of CS-10 is 28 to 30 days (idealized gestational days 42 to 44).

Studies on large collections of aborted human embryos have shown that there is a considerable variability in the developmental stage among human embryos of the same age. This means that CS-10, the period when the human embryonic heart most likely starts its pumping activity, is normally not reached at a certain day after fertilization (day 21, day 26, or day 28?), as originally proposed, but is rather reached during a certain time slot after fertilization. The exact size and position of this time slot is unknown at the present time.

The traditional methods for the estimation of the embryonic ages do not provide exact data. In order to gain precisely dated information on the development of the human embryonic heart activity, ultrasonographical studies have been conducted on human pregnancies conceived after assisted reproductive procedures (e.g., IVF) since the late 1980s. In accordance with the above-reported variability in the timing of morphological development, such studies have shown that the ultrasonographically detectable activity of the human embryonic heart normally does not start at a certain day of development but appears during a certain time slot. However, the reported time slots for the onset of ultrasonographically detectable heart activity differed considerable among the published studies. The earliest time slot ranged from 20 to 23 days after fertilization (34 to 37 gestational days), while the latest time slot ranged from 27 to 35 days after fertilization (41 to 49 gestational days). Thus, on the basis of these data, which are more than 25 years old, the first heartbeats of an individual human embryo may be expected to appear during a time span that starts at 20 days after fertilization (34 gestational days) and ends at 35 days after fertilization (49 gestational days).

In contrast to the situation in the embryological literature, direct observations on the heart activity of human embryos or fetuses ex utero were frequently documented in the non-embryological literature (e.g., journals of physiology, gynecology and obstetrics, cardiology) since the 19th century. From these articles, three reports were highlighted here since they described heart activity in living human embryos at an early stage of development. Two of them were case reports on embryos found in spontaneously aborted gestational sacs. They were published in the late 19th and early 20th century. The morphological features of the embryos described in these reports corresponded to CS-11 in both cases. The third report described a study on isolated heart specimens obtained from legal terminations of pregnancies at an age of 5 to 15 gestational weeks. In this study, regular electrical activity was already recorded in the heart specimens from embryos at a gestational age of 5 weeks. Unfortunately, information on the morphology of the embryos is missing in this report so that it is not possible to determine the CS of the embryos.

## Figures and Tables

**Table 1:** Age, greatest length and heart rate of human embryos at the onset of ultrasonographically detectable heart activity as observed in pregnancies resulting from IVF.

Post-Fertil. Age (Days)	Estimated Gest. Age (Days)	Greatest Length (mm)	Heart Rate (bpm)	Reference
25–30	39–44	2	65	[24,25]
26–32	40–46	4	80	[26]
28	42	6	~130	[27]
27–32	41–46	2	No info.	[28]
27–34	41–48	4	120	[29]
23	37	3	80	[30]
20–23	34–37	1.6	89–99	[31,32]
24	38	No info.	87	[33]

Gestational ages were estimated by adding 14 days to the known post-fertilization age. References are listed according to publication date. Note that the data from two studies were used as the basis for more than one paper. Abbreviations: bpm = beats per minute, Post-fertil. = post-fertilization, gest. = gestational, info = information.

**Table 2:** Summary of the historically most important methods used for the estimation of the post-fertilization age of human embryos and the resulting age proposals for CS-10 embryos.

Data/Methods Used for the Estimation of the Post-Fertil. Ages	Proposed Post-Fertil. Ages of CS-10 Embryos
Menstrual history/calculations according to the Reichert-His convention (~1880–1910)	13–14 days [48,52,53]
Menstrual history/calculations according to concepts of intermenstrual ovulation (~1900–1940)	21–24 days [67] 20–22 days [68]
Morphology-based staging/comparison with macaque embryos of known ovulation age (since~1940)	21–23 days [9,14]
Menstrual history/calculations according to the concept of mid-cycle ovulation (since~1940)	20–23 days [63] 26–28 days [64]
Greatest length of human embryos from the Carnegie Collection/ comparison with the embryonic growth curves obtained from first trimester sonographic examinations (since 2010)	28–30 days [47]

**Table 3:** Information on morphological features, gestational and proposed post-fertilization ages, and heart activity of human CS-10 embryos as provided in case reports published in journals/books of embryology, teratology, or anatomy.

Report and “Name of Embryo”	Number of Somite Pairs	Greatest Length (mm)	Proposed Post-Fertil. Age (Days)	Gest. Age (Days)	Heart Activity
[49] “SR”	6–7	2.2	~14 ^RH^	-	No info.
[89] von Spee	7	2.69	14 ^RH^	42	No info.
[17,18] Eternod “Du Ga”	7	2.11	13–14 ^RH^	-	No info.
[90] “Klb”	5–6	1.8	12–14 ^RH^	-	No info.
[67] Dandy	7	2	13–14 ^RH^ 24 ^IM^	43	No info.
[84] “H 98“	8	1.27		38	No info.
[68] “H87”	7–8	2	21 ^IM^	-	No info.
[91] Veit & Esch	8	2.5	-	40	No info.
[92] Payne	7	2.17	-	-	No info.
[93] “H279”	4	2.5	-	28 or 35	No info.
[93] “H197”	12	2.08	-	44	No info.
[93] “H392”	11	3.6	-	45	No info.
[94] Sternberg	4	2.3	-	-	No info.
[95] “Da2”	10	-	-	-	No info.
[85] “H10”	10	3	-	42	No info.
[96]; “Bi II”	4–5	-	-	-	No info.
[96]; “Bi III”	4–5	-	-	-	No info.
[96,97] “Bi XI”	10	2.2	-	-	No info.
[98] Politzer	7	~3	-	-	No info.
[99] West	8	-	-	-	No info.
[100] “H.Schm._2_.”	10	2.4	20 ^C^	29	No info.
[101,102] Litzenberg	12	-	-	47	No info.
[103] Orts Llorca	4	-	-	-	No info.
[86] Baxter & Boyd	10	-	28 ^MC^	42	No info.
[104] Arey & Henderson	6	-	-	-	No info.
[87] Holmdahl	11	1.7	-	47	No info.
[105] Streiter	7	1.93	21–22 ^MC^	-	No info.
[106] Schenk	5			-	No info.
[14] “2795”	4–5	2	22 ± 1 St	-	No info.
[14] “H1404”	7–8	2.83	22 ± 1 St	-	No info.
[14] “8244”	6	1.55	22 ± 1 St	-	No info.
[14] “H637”	12	-	22 ± 1 St	-	No info.
[107] “No. 103”	5	-	-	-	No info.
[107] “No. 101”	8	-	-	-	No info.
[88] “No. 5074”	12	-	-	-	No info.

Reports are listed according to publication date. The post-fertilization ages, proposed by the authors of the reports, were estimated as follows: RH = calculation according to the Reichert-His convention; IM = calculations according to diverse concepts of intermenstrual ovulation; MC = calculations according to the concept of mid-cycle ovulation; C = coital age; St = estimations according to Streeter (matching with macaque embryos]. Abbreviations: fertil. = fertilization; gest. = gestational; info. = information.

**Table 4:** List of reports or studies on human embryo collections checked for information on embryonic heart activity (HA).

Name of Collection, Report	Number of Embryos	* Greatest Length or CS of Embryos	Number of CS-10 Embryos	Greatest Length of CS-10 Embryos (mm)	Proposed Post-Fertil. Age of CS-10 Embryos (Days)	Gestational Age of CS-10 Embryos (Days)	Number of Embryos with HA	* Length or CS of Embryos with HA
Carnegie-Coll., [108]	533	* 1–220 mm	?	-	-	-	22	* 11–96 mm
Carnegie-Coll., [109]	2500	-	?	-	-	-	1	* 21 mm
Carnegie-Coll., [115]	483	6–23	11	2.16 (mean)	-	-	0	-
Kyoto-Coll. [64]	1213	12–23	0	-	-	-	0	-
Kyoto-Coll. [116]	90	7–13	8	1.5–2.7	27 (mean)	41 (mean)	0	-
Kyoto-Coll. [117]	37	6–11	10	-	22–23	-	0	-
Edinburgh-Coll., [118]	310	7–23	13	2.9 ± 0.12	-	45.2 ± 1.2	0	-
Newcastle-Coll., [119]	60	10–22	1	-	-	-	0	-
Carnegie-Coll., [120]	494	2–23	20	2 (mean)	27 (mean)	-	0	-
Carnegie-Coll., [47]	407	1–23	13	1.5–3.6	28–30	-	0	-

Reports/studies are listed according to publication date. In the first 2 reports, no information was provided about the CS of the embryos. Instead of it, information was given about the greatest length of the embryos (*). Abbreviations as used before.

**Table 5:** List of studies on human embryonic hearts checked for information on heart activity.

Study/Source of Specimens	Number of Specimens	Range of CS	Number of CS-10 Embryos	Greatest Length of CS-10 Embryos (mm)	Proposed Post-Fert. Age at CS-10 (Days)	Number of Embryos with HA	CS of Embryos with HA
[121]/Carnegie-Coll.	58	10–23, + 4 fetuses	3	2–2.5	-	0	-
[10]/Carnegie-Coll. + published cases	11	9–11	7	1.8–3.09	-	0	-
[70]/Carnegie-Coll. + own specimens	24	9–15	9	-	-	0	-
[122]/Carnegie-Coll.	54	9–23	4	-	-	0	-
[111]/Carnegie-Coll.	351	9–23	12	2.2 ± 0.8	-	10	17–22
[123]/own specimens	11	10–16	2	-	22–23	0	-
[124]/own specimens	29	10–16	3	-	22 ± 1	0	-
[125]/own specimens	300	10–16	?	-	-	0	-
[126]/own specimens	?	10–16	?	-	-	0	-

This list includes only those studies in which hearts from CS-10 embryos have been examined. Studies are listed according to publication date. Abbreviations as used before.

**Table 6:** List of reports/studies on human embryonic or fetal heart activity found in journals or books of physiology, gynecology and obstetrics, reproductive medicine, cardiology, etc.

Report	Number of Specimens	Greatest Length of Embryos/ Fetuses (mm)	CS	Proposed Post-Fert. Age	Gest. Age (Weeks)	Kind of Specimens	Kind of Study	Spontaneous Heart Rate (bpm]
[138]	10	-	-	-	4.5–10	myocardial cells	Electro-physiol.	60–150 ^37 °C^
[147]	165	-	-	-	5–15	hearts	Heart rate recordings	157–180 ^37 °C^
[131,132]	1	2.5	11	14 days ^RH^	6	embryo	Case report	90 ^RT^
[127]	1	-	11	18–20 days	-	embryo	Case report	Occasional ^RT^
[133]	1	4	13 or 14	47–50 days	-	embryo	Case report	70 ^RT^
[135]	5	6–60	-	2 weeks–2.5 months	-	2 embryos + 3 fetuses	ECG	Occasional ^RT^
[139]	37	-	-	-	7–12	hearts	Electro-physiol.	50–132 ^37 °C^
[136]	1	23	-	7.5 weeks	9.5	embryo	ECG	40–80
[145]	2	30 + 30	-	-	9 + 10	hearts	Experiments	140 ^37 °C^
[134]	11	-	-	-	9.5–25	fetuses	ECG	48–100 ^RT^
[137]	11	55–170	-		10–18	fetuses	ECG	No info.
[129]	1	75	-		13	fetus	Case report	No info.
[141]	5	100–165	-	-	12–16	hearts	Electro-physiol.	No info.
[140]	14	-	-	-	12–22	hearts	Electro-physiol.	142 ^37 °C^
[146]	17	-	-	-	12–22	hearts	Experiments	142 ^37 °C^
[143]	1	100	-	13 weeks	-	heart	Experiments	157 ^37 °C^
[128]	1	100	-	-	16	fetus	Case report	No info.
[130]	1	-	-	-	15	fetus	Case report	30 ^RT^
[144]	9	-	-	-	16–24	hearts	Experiments	No info.
[142]	2	-	-	-	24	hearts	Experiments	40 + 100 ^37 °C^

Reports are listed according to the age of the embryo(s) or fetus(es). Note that the CS were not provided in the original reports but were determined, by the author of the present paper, on the basis of the reported morphological features or on the basis of photographs. Abbreviations: RT = room temperature; other abbreviations as used before.
